# How Healthy Lifestyle Factors at Midlife Relate to Healthy Aging

**DOI:** 10.3390/nu10070854

**Published:** 2018-06-30

**Authors:** Nathalie Atallah, Moufidath Adjibade, Hélène Lelong, Serge Hercberg, Pilar Galan, Karen E. Assmann, Emmanuelle Kesse-Guyot

**Affiliations:** 1 Nutritional Epidemiology Research Team (EREN), Epidemiology and Statistics Research Center, U1153 National Institute of Health and Medical Research (INSERM), U1125 National Institute for Agricultural Research (INRA), National Conservatory of Arts and Crafts (CNAM), Paris 13 University, Sorbonne Paris Cité COMUE, F-93017 Bobigny, France; nathalie.atallah@agroparistech.fr (N.A.); m.adjibade@eren.smbh.univ-paris13.fr (M.A.); helene_lelong@hotmail.com (H.L.); s.hercberg@eren.smbh.univ-paris13.fr (S.H.); p.galan@eren.smbh.univ-paris13.fr (P.G.); k.assmann@eren.smbh.univ-paris13.fr (K.E.A.); 2 AP-HP, Diagnosis and Therapeutic Center, Faculty of Medicine, Hôtel-Dieu Hospital, Paris-Descartes University, F-75004 Paris, France; 3 Département de Santé Publique, Hôpital Avicenne, F-93017 Bobigny, France

**Keywords:** smoking, diet, physical activity, alcohol

## Abstract

With increasing life expectancies worldwide, it is an important public health issue to identify factors that influence the quality of aging. We aimed to investigate the individual and combined roles of lifestyle factors at midlife for healthy aging (HA). We analyzed data from 2203 participants of the French ”Supplémentation en Vitamines et Minéraux Antioxydants” (SU.VI.MAX) cohort aged 45–60 years at baseline (1994–1995), and assessed the combined impact of lifestyle factors (weight, smoking status, physical activity, alcohol consumption, and diet) on HA (absence of chronic diseases and function-limiting pain, good physical and cognitive functioning, functional independence, no depressive symptoms, and good social and self-perceived health) with a five-component healthy lifestyle index (HLI). Relative risks (RR) and 95% confidence intervals (CIs) were estimated using a robust-error-variance Poisson regression. Approximately 39% of our sample aged healthily. After adjustment for potential confounders, a one-point increase in HLI was related to an 11% higher probability of HA (95% CI = 6%, 16%; *p* < 0.001). The proportions of HA attributable to specific factors based on the “population attributable risk” concept were 7.6%, 6.0%, 7.8%, and 16.5% for body mass index (BMI), physical activity, diet quality, and smoking status, respectively. This study highlights the importance of healthy lifestyle habits at midlife for the promotion of good overall health during aging.

## 1. Introduction

Life expectancy steadily increased over the past few decades, and this surge is expected to continue in industrialized countries [1], as well as in developing countries [2]. Moreover, the observed inter-individual variability in terms of health status during aging [3] led to numerous theories on the causes of aging [2], and to the development of a concept known as “successful” or “healthy” aging (HA) [4]. A turning point in this field of research was the model proposed by Rowe and Kahn, who defined HA as having a low risk of disease and disease-related disability, high cognitive and physical functional capacity, and being actively engaged with life [3]. While a consensual definition of HA is not agreed upon, a number of potentially modifiable lifestyle factors that could influence quality of aging were identified, such as smoking status, physical activity, and diet [5,6]. Most studies investigating the relationship between modifiable lifestyle factors and HA focused on the role of a single lifestyle factor. However, since individuals do not adhere to lifestyle behaviors in isolation, it is essential to take an overall approach [7]. In an epidemiological context, adherence to a healthy lifestyle can be evaluated through the construction of a priori scores. A number of studies already examined the relationship between a healthy lifestyle index (HLI) and various health outcomes [8,9,10]. To the best of our knowledge, studies evaluating the impact of lifestyle factors on HA are scarce. One cross-sectional study directly evaluated the links between a combination of modifiable lifestyle factors and HA with an a priori score [11]. Their results demonstrated that adherence to the HLI was positively associated with HA, and the likelihood of HA increased with each positive lifestyle behavior present in the index. Another study [12] conducted in the Whitehall population explored the combined effect of healthy lifestyle factors on HA, but limited data was available for the estimation of nutritional quality of the diet, and body mass index (BMI) was not accounted for. However, no study directly examined the long-term effect of midlife lifestyle factors with accurate dietary data on HA over a sufficiently long period of time. Hence, the aim of our study was to determine the association between combined and individual lifestyle factors and HA status after a follow-up period of 13 years in a large cohort of French adults. Moreover, we aimed to compare the different contributions of these lifestyle factors to HA using the “population attributable risk” (PAR) concept.

## 2. Design and Methods

### 2.1. Study Design and Participants

The “Supplémentation en Vitamines et Minéraux Antioxydants” (SU.VI.MAX, 1994–2002) study was a randomized double-blind, placebo-controlled, primary prevention trial. A total of 12,741 volunteers living in France, aged from 35–60 years for women and from 45–60 years for men at baseline, participated in this study. The study was previously described in greater detail [13]. Five years after the end of the SU.VI.MAX trial, an observational follow-up trial known as SU.VI.MAX 2 (2007–2009) was conducted. The SU.VI.MAX 2 study included 6850 individuals from the initial SU.VI.MAX study who agreed to participate on a voluntary basis. They were invited to complete clinical and neuropsychological tests, as well as questionnaires, in 2007–2009 [14]. Both studies were operated in accordance with the guidelines from the Declaration of Helsinki, and procedures implicating human subjects were approved by the ethics committee for studies with human participants of the Cochin Hospital, Paris (CCPPRB (Comité consultatif de protection des personnes dans la recherche biomédicale) numbers 706 and 2364 for SU.VI.MAX and SU.VI.MAX 2, respectively), and the “Commission Nationale Informatique et Liberté” (CNIL numbers 334 641 and 907 094, respectively). Informed written consent was also obtained from the participants. 

### 2.2. Data Collection

Sociodemographic, lifestyle, and dietary data (1994–1996):

Information on sex, date of birth, education (primary, secondary, or post-secondary), socio-professional category (homemaker, manual worker, employer, or managerial staff/intellectual profession), living status (alone or cohabiting), smoking status (never smoked, or former or current smoker), physical activity (0–30 min/d of walking, 30–60 min/d of walking, or ≥60 min/d of walking), and medical treatment was collected via self-administered questionnaires. BMI (kg/m^2^) was calculated from anthropometric data by trained personnel.

During the SU.VI.MAX study, participants filled out 24-h dietary records through computerized questionnaires every two months, which covered all seasons and days of the week. They were presented with an instruction manual containing >250 validated food photographs and seven different portion sizes to choose from. If energy intake was <418.4 kJ/d (<100 kcal/d) or >25,104 kJ/d (>6000 kcal/d), the record was considered invalid. An additional measure to account for energy underreporting consisted of excluding men reporting <3347 kJ/d (<800 kcal/d) and women reporting <2092 kJ/d (<500 kcal/d) across ≥1/3 of records. Seafood and alcohol are known to be consumed rather infrequently by many individuals; hence, they were assessed with specific questionnaires. Mean food and nutrient intakes were calculated from the valid 24-h dietary records from the first two years of the study using a food composition table referencing >900 foods, estimating a typical diet during adulthood.

Health data and definition of “healthy aging” status (1994–2009):

The collection of cardiovascular disease (CVD) and cancer records was described extensively in previous studies [15]. In brief, the data on these events was obtained from questionnaires sent every six months to the participants, then validated by an expert committee with a review of medical records on the basis of the 10th International World Health Organization Classification of Diseases (WHO International Classification of Diseases). Baseline and follow-up diabetes statuses (fasting blood glucose ≥7 mmol/L, or use of anti-diabetic medication) were also identified.

The definition of HA was mainly based on the concept introduced by Rowe and Kahn [3]. It was defined as living in the absence of major chronic diseases (cancer, CVD, or diabetes) during follow-up assessments, having no limitations in instrumental activities of daily living (IADL), and having no function-limiting pain, depressive symptoms, or health-related limitations in social life. It also included having good physical and cognitive functioning, as well as good overall self-perceived health. The components of HA were defined as binary variables, and more details on its construction and the assessments can be found in a previous publication [16] and in Supplementary Materials Table S1.

### 2.3. Data Treatment and Statistical Analysis

The development and computation of the healthy lifestyle index (ranging from 0 to 5) are described in Tables S1 and S2. It is composed of five binary components pertaining to BMI, smoking status, alcohol consumption, diet quality, and physical activity.

### 2.4. Study Sample Selection: 

Information on health status in 2007–2009 was available for participants who voluntarily agreed to participate in the SU.VI.MAX 2 study (*n* = 6850). Only those aged from 45–60 years old and free of chronic diseases upon inclusion in the SU.VI.MAX study were included in our sample. Exclusion of those with missing data on HA status, on the HLI, or on co-variables led to a final study sample of 2203 individuals (Figure S1).

### 2.5. Statistical Analysis

Characteristics of the participants were described as mean ± standard deviation (SD) or percentages across the various HLI categories. The *p*-values referred to linear contrast tests or Mantel–Haenszel Chi^2^ trend tests. Since the frequency of individuals with a score of 0 was minimal (*n* = 20), individuals with scores of 0 and 1 were grouped together throughout the manuscript. Poisson regressions with robust-error variance were applied to determine the association between the HLI (modeled as categories and as a continuous variable) and HA. For categories, *p*-values for linear trends were estimated using linear contrast tests. Model 1 was adjusted for baseline age and gender, and Model 2 was additionally adjusted for marital status, education, occupational status, supplementation group, number of 24-h dietary records, follow-up time, and energy intake. In order to confirm that the observed associations were not due to the effect of a single HLI component, a sensitivity analysis was conducted using modified HLIs in which every component was removed one by one. These modified HLIs were modeled categorically and as continuous independent variables, and were further adjusted for the removed component. A supplemental model (Model 3) was created, which was similar to Model 2, but the removed binary component was replaced by the corresponding continuous indicator. For example, instead of inserting the BMI as a binary component of the HLI, continuous BMI was included in the model. Moreover, associations between the HLI and HA were tested for individuals who had filled out ≥6 24-h dietary records. As there were no relationships between gender and HLI, and between the supplemented and placebo groups, all analyses were carried out simultaneously. 

A modified version of the population attributable risk (PAR) concept called “mPAR” was calculated for every lifestyle factor, while assuming that the association between each lifestyle factor in the HLI and HA was causal. This mPAR can be interpreted in the same way as PAR estimates, except that higher mPAR estimates indicate better health outcomes (i.e., an increased probability of aging healthily) in relation to specific exposure factors. Thus, the mPAR corresponds to the population attributable increase in HA probability. 

The following equation [17] was used to determine the mPARs for each component of the HLI: *ρ* × $\frac{RR-1}{RR}$, where *ρ* denotes the prevalence of healthy agers showing a healthy behavior, and RR denotes the relative risk associated with that healthy behavior. The mPAR was also calculated for individuals who practiced at least half of the behaviors included in the HLI (HLI > 2). Confidence intervals (CI) were estimated through the use of a bootstrap method (500 estimations). All analyses were performed using the SAS Enterprise Guide 7.1 software (SAS Institute Inc., Cary, NC, USA). 

## 3. Results

A total of 2203 participants (45.7% women) were included in the present analysis. Mean (SD) age at baseline was 49 years (6), and 39.4% of participants presented HA upon follow-up. Baseline characteristics according to HLI scores are shown in Table 1. Participants with the highest score (HLI = 5) were most often women, non-smokers, employers, and those who were physically active. Moreover, they had lower energy intakes, consumed more plant proteins and carbohydrates, and less alcohol, and exhibited a higher quality of diet.

A one-point increase in the HLI was associated with an 11% (95% CI) increase in the probability of HA (Table 2). A higher HLI score in the fully adjusted model (RR HLI_5 versus 0/1_ = 1.41; 95% CIs = 1.10, 1.80; *p*-value < 0.001) resulted in a higher likelihood of HA (Table S3). The modified HLIs also led to significant results in terms of HA (Model 2), except in the case of the HLI without physical activity considered as a categorical variable (Table S3). The association was strongest for the HLI without alcohol. When the removed components were entered as continuous independent variables (Model 3), the results were similar.

Table 3 represents the association between the HLI and various specific components of our HA definition. In the fully adjusted model, an increase in the HLI score was significantly associated with the absence of chronic diseases (*p* = 0.001), good physical functioning (*p* < 0.001), good overall self-perceived health (*p* = 0.005), no function-limiting pain (*p* < 0.001), no limitations in IADL (*p* = 0.002), and no depressive symptoms (*p* = 0.006). On the other hand, no significant association was observed between the HLI and the absence of health-related limitations in social life or good cognitive functioning.

The calculated mPARs can be found in Table 4. The obtained mPARs showed that 7.6% of cases of HA were attributable to having a healthy BMI, 6.0% of cases to practicing physical activity, 7.8% of cases to having a healthy diet, and 16.5% of cases to not smoking. The mPAR obtained for alcohol consumption was non-interpretable. Finally, 14.6% of cases of HA were attributable to practicing at least three healthy behaviors (HLI > 2). Table S4 shows the results of the sensitivity analysis performed on cases with ≥6 24-h dietary records; the main results were unchanged.

## 4. Discussion

In this large cohort of middle-aged French adults, higher scores on the HLI were associated with a higher probability of HA after adjustment for a wide range of potential confounders, and in the sensitivity analysis. The observed relationship was linear, indicating that, when practiced in combination, they have a greater impact on health. Moreover, a score of 5 on the HLI was associated with the majority of the HA components. 

We found that among those individuals who aged healthily, 7.6% of cases of HA were attributable to a healthy weight (18.5 < BMI < 25), and 7.8% to high dietary quality. These results are in line with findings from previous epidemiological studies on the association of BMI and diet quality with HA. In particular, in an analysis of data from the British White Hall II study [18], a negative association was observed between BMI and HA. Since obesity is often associated with numerous chronic diseases, such as cardiovascular disease, type 2 diabetes, and certain cancers [19], it can lead not only to a decrease in quality of life, but also to premature death. This association was confirmed in a 20-year follow-up study conducted on data from the National Health and Nutrition Examination Survey (NHANES I) and its Epidemiologic Follow-up study (NHEFS) [20]. Concerning diet quality, a previous examination disclosed that individuals with high overall dietary quality as measured by the Alternative Healthy Eating Index-2010 (AHEI-2010) and the alternate Mediterranean diet score had higher odds of HA when compared with those with low overall dietary quality [21]. Moreover, 6% of “cases” of HA in our study population were attributable to being physically active. This is in line with results from a study based on data from the English Longitudinal Study of Aging population, in which practicing moderate to vigorous physical activity at least once a week was significantly associated with HA [22]. Beyond the well-known beneficial role of physical activity concerning chronic diseases [23], this could be explained by the role that physical activity plays with respect to cognitive functioning [24] and physical functioning [25]. 

Furthermore, 16.5% of “cases” of HA in our study sample were attributable to not smoking or quitting smoking, and 14.6% of cases were related to practicing at least three of the healthy behaviors included in the HLI. Hence, not being a smoker appeared to have a stronger positive association with HA than the combination of any three different healthy behaviors, which is in line with the fact that smoking is a major risk factor for many serious diseases. Indeed, smoking is implicated in the etiology of cardiovascular, neoplastic, and respiratory diseases through multiple mechanisms [26]. Some of these mechanisms include an increase in oxidative stress via not only the production of free radicals, but also via the reduction of antioxidant defense systems. The key role of avoiding smoking for HA observed in our study underlines the need for more effective public health strategies aimed at decreasing the proportion of smokers among middle-aged individuals and in the general population. Finally, no meaningful mPAR estimate was obtained in the case of alcohol consumption. This could be because the cut-off values chosen for alcohol consumption in our study might not be adapted to the context of HA. It is also important to note that we did not discriminate between moderate drinkers and alcohol abstainers who might also practice other specific health behaviors. Moreover, it is important to note the absence of heavy drinkers in our study. 

Overall, studies on the association of a combination of lifestyle factors with HA are rare. To the best of our knowledge, only two studies evaluated the relationship between healthy lifestyle factors in combination with HA [11,12]. In the first study by Sowa et al., data from 5139 men and 5909 women aged 50 years or more were collected from the fourth wave of the Survey of Health, Aging, and Retirement in Europe (SHARE) population for a period of two years. They concluded that physical activity, a healthy diet based on fruits and vegetables, a high consumption of liquids, regular meals, and abstinence from smoking were all positively associated with HA. Despite the differences between that study and our study concerning definitions of both the HLI (components and scoring) and HA, the findings of both studies reinforce the assumption that healthy behaviors at midlife can have an impact on aging. 

In the study performed on data from the Whitehall II cohort, they evaluated the association between individual and combined lifestyle factors at midlife with HA [12]. The findings were similar to those in our study, since they demonstrated that never smoking, moderate alcohol consumption, physical activity, and eating fruits and vegetables were all linked to higher odds of HA. Moreover, similarly to our results, the benefits in terms of HA increased linearly for each additional compliance with a healthy behavior. However, their calculated PARs, which correspond to the mPARs in our study, were much larger than the ones we obtained. This could be explained not only by differences in adjustment factors (much more numerous in our study), but also by differences in HA definitions. 

A number of limitations can be identified in this study. Firstly, individuals included in the SU.VI.MAX study might not be representative of the French population. Furthermore, dichotomization of each component of the HLI according to specific cut-offs may influence the results of this study. However, in the case of alcohol consumption and diet quality, various cut-offs were tested, and the choice of cut-offs did not affect our main results. An important strength of our HA definition is the inclusion of objective factors, as well as more subjective factors, rendering our definition more complete. Moreover, a sensitivity analysis excluding individuals with less accurate dietary data confirmed the robustness of the association found between the HLI and HA. Other strengths of this study include the long follow-up period (13 years) and longitudinal design, as well as the use of midlife factors, thus providing a life-course perspective. Additionally, our study had a very comprehensive overall approach since it took into account diet as a whole [27]. Finally, mPARs are measures of health impact [28], and can give information on the proportion of HA that can be attributed to adhering to a specific healthy lifestyle factor. They can, thus, be of great value for the implementation of public health policies regarding the achievement of good health in the elderly.

## 5. Conclusions

In conclusion, this study highlights the importance of promoting healthy lifestyle habits at midlife in order to age in good health. Even following only a selection of various healthy behaviors can have important public health impacts, and larger benefits can be expected with an increasing adherence to an overall healthy lifestyle. Moreover, calculated mPARs indicated an individual contribution of each healthy lifestyle factor to HA, except for alcohol consumption, with smoking status as the most prominent factor. However, further interventional studies are needed to confirm these findings.

## Figures and Tables

**Table 1:** Baseline characteristics of the study population across classes of healthy lifestyle index (HLI; *N* = 2203) ^a^.

Characteristics	HLI = 0 or 1		HLI = 2		HLI = 3		HLI = 4		HLI = 5		*p* ^b^
	**184**		**498**		**713**		**594**		**214**		
**Gender**	%	*n*	%	*n*	%	*n*	%	*n*	%	*n*	<0.001
Men	83	153	59	294	53	375	48	282	43	92	
Women	17	31	41	204	47	338	52	312	57	122	
**Education**											0.87
Primary	22	40	21	107	20	145	20	119	23.4	50	
Secondary	37	69	41	202	42	296	38.2	227	39.7	85	
Post-secondary	41	75	38	189	38	272	41.8	248	36.9	79	
**Living status**											0.16
Alone	10	18	11	54	15	106	12	70	15	32	
Cohabiting	90	166	89	144	85	607	88	524	85	182	
**Occupational status**											0.02
Homemaker	2	4	6	28	7	48	9	55	10	22	
Manual worker	12	22	6	28	6	42	5	27	5	10	
Employer	48	88	57	285	57	404	54	322	55	118	
Manager staff	38	70	31	157	30	219	32	190	30	64	
**Physical activity**											<0.001
0–30 min/day	95	174	78	388	60	430	34	204	0	0	
30–60 min/day	3	6	10	51	21	148	34	199	49	105	
≥60 min/day	2	4	12	59	19	135	32	191	51	109	
**Smoking status**											<0.001
Non-smokers	22	41	42	211	53	380	59	353	62	133	
Former smokers	38	70	39	195	40	287	38	224	38	81	
Current smokers	40	73	19	92	7	46	3	17	0	0	
	**Mean (SD)**		**Mean (SD)**		**Mean (SD)**		**Mean (SD)**		**Mean (SD)**		
Age at baseline (years)	52.3 (4.4)		51.6 (4.5)		51.9 (4.6)		51.9 (4.5)		52.0 (4.7)		0.60
Body mass index (kg/m^2^)	27.0 (2.8)		25.5 (3.4)		24.4 (3.2)		23.4 (2.6)		22.4 (1.6)		<0.001
Energy intake (kcal)	2478 (633)		2325 (607)		2190 (621)		2113 (587)		2059 (584)		<0.001
%Proteins	16 (2.6)		16.4 (2.5)		16.9 (2.7)		16.7 (2.5)		16.8 (2.3)		0.16
%Animal protein	12.4 (2.9)		12.1 (2.6)		12.4 (2.9)		12.0 (2.7)		11.9 (2.6)		0.05
%Plant proteins	4.1 (0.8)		4.3 (0.7)		4.5 (0.8)		4.7 (0.8)		4.9 (0.9)		<0.001
%Carbohydrates	35.3 (7.2)		38.1 (6.2)		39.4 (6.2)		41.2 (6.0)		43.1 (6.4)		<0.001
%Lipids	37.3 (5.1)		37.5 (4.9)		37.9 (4.8)		37.6 (5.0)		37.2 (5.4)		0.88
%SFA	15.1 (2.5)		15.6 (2.5)		15.5 (2.6)		15.4 (2.7)		15.1 (2.8)		0.42
%PUFA	5.7 (1.6)		5.5 (1.4)		5.7 (1.5)		5.8 (1.4)		5.8 (1.6)		0.13
%MUFA	14.2 (2.3)		14.1 (2.1)		14.3 (2.1)		14.2 (2.2)		14.1 (2.3)		0.54
Alcohol (g)	36.2 (18.5)		24.0 (16.5)		16.8 (17.0)		10.3 (12.8)		4.0 (7.1)		<0.001
mPNNS-GS without alcohol	5.5 (1.1)		6.2 (1.4)		6.7 (1.4)		7.3 (1.3)		7.9 (1.1)		<0.001

HLI: healthy lifestyle index; SFA: saturated fatty acids; PUFA: polyunsaturated fatty acids; MUFA: monounsaturated fatty acids; mPNNS-GS: modified “Programme National Nutrition Santé” guideline score. ^a^ Values are given as mean ± SD or % as appropriate; ^b^ *p*-values for linear contrast tests or Mantel–Haenszel tests.

**Table 2:** Association between the original and modified versions of the continuous HLI and healthy aging (*N* = 2203) ^a.^

	Continuous HLI	
	RR 95%CI	*p* ^b^
**HLI original**		
Model 1 ^c^	1.12 (1.07, 1.17)	<0.001
Model 2 ^d^	1.11 (1.06, 1.16)	<0.001
**Without BMI**		
Model 1 ^e^	1.11 (1.05, 1.17)	<0.001
Model 2 ^f^	1.10 (1.05, 1.16)	<0.001
Model 3 ^g^	1.10 (1.04, 1.16)	<0.001
**Without physical activity**		
Model 1 ^e^	1.12 (1.06, 1.18)	<0.001
Model 2 ^f^	1.09 (1.03, 1.16)	0.002
Model 3 ^g^	1.09 (1.03, 1.16)	0.002
**Without smoking status**		
Model 1 ^e^	1.11 (1.05, 1.16)	<0.001
Model 2 ^f^	1.10 (1.05, 1.15)	<0.001
Model 3 ^g^	1.10 (1.04, 1.15)	<0.001
**Without alcohol**		
Model 1 ^e^	1.17 (1.10, 1.23)	<0.001
Model 2 ^f^	1.15 (1.09, 1.21)	<0.001
Model 3 ^g^	1.15 (1.09, 1.21)	<0.001
**Without diet**		
Model 1 ^e^	1.10 (1.04, 1.16)	0.001
Model 2 ^f^	1.10 (1.04, 1.16)	0.001
Model 3 ^g^	1.09 (1.04, 1.16)	0.001

HLI: healthy lifestyle index; BMI: body mass index; ^a^ values are given as relative risk (RR) with 95% confidence intervals (CIs); ^b^ *p*-values for the HLI as a continuous variable; ^c^ adjusted for age and gender; ^d^ adjusted for age, gender, marital status, education, occupational status, supplementation group, number of 24-h dietary records, follow-up time, and energy intake; ^e^ adjusted for all variables in Model 1 and removed components; f adjusted for all variables in Model 2 and removed components; ^g^ adjusted for all variables in Model 2, and more precisely removed components.

**Table 3:** HLI in relation to each component of healthy aging (*N* = 2203) ^a^.

	Continuous HLI	*p* ^b^
**No chronic diseases**		
Model 1 ^d^	1.03 (1.01, 1.04)	0.002
Model 2 ^e^	1.03 (1.01, 1.04)	0.001
**Good physical functioning**		
Model 1 ^d^	1.07 (1.04, 1.09)	<0.001
Model 2 ^e^	1.06 (1.04, 1.09)	<0.001
**Good cognitive functioning**		
Model 1 ^d^	0.99 (0.98, 1.01)	0.57
Model 2 ^e^	0.99 (0.97, 1.01)	0.37
**No limitations in IADL**		
Model 1 ^d^	1.02 (1.01, 1.03)	<0.001
Model 2 ^e^	1.02 (1.01, 1.03)	0.002
**No depressive symptoms**		
Model 1 ^d^	1.02 (1.01, 1.04)	0.005
Model 2 ^e^	1.02 (1.01, 1.04)	0.006
**Good overall self-perceived health**		
Model 1 ^d^	1.01 (1.00, 1.02)	0.005
Model 2 ^e^	1.01 (1.00, 1.02)	0.005
**No health-related limitations in social life**		
Model 1 ^d^	1.01 (1.00, 1.02)	0.07
Model 2 ^e^	1.01 (1.00, 1.02)	0.15
**No function-limiting pain**		
Model 1 ^d^	1.03 (1.01, 1.04)	<.001
Model 2 ^e^	1.03 (1.01, 1.04)	<.001

HLI: healthy lifestyle index; IADL: instrumental activities of daily living; ^a^ values are given as RR (95% CIs); ^b^ *p*-values for the HLI as a continuous variable; ^d^ adjusted for age and gender; ^e^ adjusted for age, gender, marital status, education, occupational status, supplementation group, number of 24-h dietary records, follow-up time, and energy intake.

**Table 4:** Modified population attributable risks (mPARs) according to individual lifestyle factors.

	“Healthy Agers” Also Presenting the Respective Lifestyle Factor ^a^	%mPAR (95% CI)
Healthy weight	572	7.6 (2.0, 13.3)
Being physically active	424	6.0 (2.2, 9.5)
High diet quality	521	7.8 (2.9, 12.5)
Low alcohol consumption	454	−1.1 (−5.5, 3.9)
Not smoking	794	16.5 (3.5, 28.9)
HLI > 2	806	14.6 (7.7, 21.2)

BMI: body mass index; HLI: healthy lifestyle index. ^a^ Individuals corresponding to the definition of healthy aging (HA) who also presented the specific healthy lifestyle behavior.

## Supplementary Materials

The following are available online at http://www.mdpi.com/2072-6643/10/7/854/s1, Figure S1: Flowchart of the selection process, Table S1: Definition of the HLI, Table S2: Criteria for healthy aging, Table S3: Association between the HLI (by scores) and healthy aging, Table S4: HLI in relation to healthy aging for individuals with ≥6 24-h records, Text S1: Computation of the Healthy Lifestyle Index.
