# Supplementary material for: How Healthy Lifestyle Factors at Midlife Relate to Healthy Aging
Source: Nutrients. 2018 Jun 30;10(7):854. doi: 10.3390/nu10070854 (PMC6073192; doi:10.3390/nu10070854)
Supplement: Supplementary file 1 [file nutrients-10-00854-s001.zip › Supplementary Figure 1.docx]

**Supplementary Figure 1. Flowchart of the selection process**

1,277: Excluded due to one or more missing variables contributing to the definition of “healthy aging”

Participants of the SU.VI.MAX study
N=13,017

6,167: Excluded because not part of the SU.VI.MAX 2 study

6,850: Included in the SU.VI.MAX 2 study observational follow-up study

5,583: Within desired age-range of 45-60 years at baseline

1,267: Excluded because age outside the desired range

5,243: Free of major chronic diseases at baseline

340: Excluded due to diabetes at baseline or event of ischemic disease or cancer before baseline

2,255: Available information for the HLI

1,711: Excluded due to one or more missing variables for the determination of the HLI

2,203: Available information on all covariables

3,966: Available information for the determination of “healthy aging”

52: Excluded due to one or more missing covariables
