# Supplementary material for: How Healthy Lifestyle Factors at Midlife Relate to Healthy Aging
Source: Nutrients. 2018 Jun 30;10(7):854. doi: 10.3390/nu10070854 (PMC6073192; doi:10.3390/nu10070854)
Supplement: Supplementary file 1 [file nutrients-10-00854-s001.zip › Supplementary material.docx]

**Supplementary material:**

**Supplementary Table 1. Definition of the HLI**

| Component | Points | Description |
| --- | --- | --- |
| Healthy weight | 0  1 | Overweight or obesity BMI ≥ 25 18.5 ≤ BMI <25 |
| Smoking status | 0  1 | Smoker Former or non-smoker |
| Physical activity | 0  1 | Physically inactive Moderate to high physical activity (>30 min/d brisk walking or equivalent) |
| Alcohol consumption | 0  1 | > 12g/d for women or >24g/j for men ≤ 12g/d for women or ≤ 24g/j for men |
| Diet quality | 0  1 | ≤ 6,5 points on the mPNNS-GS > 6,5 points on the mPNNS-GS |

Abbreviation: mPNNS-GS, Modified Programme National Nutrition Santé-Guideline Score
PNNS-GS measures adherence to the French recommendations for consumption of fruits and vegetables, starchy foods, whole grains, dairy products, meats, seafood, added fat, sweets, water, soda, and salt. It also penalizes for excessive energy consumption. In our study a modified score excluding physical activity and alcohol has been considered (maximum score=12.5).
**Supplementary Text 1. Computation of the Healthy Lifestyle Index**

The Healthy Lifestyle Index (HLI) was based on a previous study (1) and included risk factors often associated with age-related chronic diseases and functioning disorders. The index was constituted of five components, each allocated a binary score. A score of 1 was attributed to favorable behaviors, while a score of 0 was allocated when conditions were not met. The final score was obtained by summing the scores of each individual component. Thus, the HLI ranged from 0 (for least healthy) to 5 (for most healthy). The index took into account having a healthy weight (18.5<BMI<25 according to the WHO cutoff points)(2), being a non-smoker (former or non-smoker), being physically active (an activity equivalent to at least 30 min of brisk walking per day), being a moderate drinker (up to 2 standard drinks/d for men and up to 1 standard drink/d for women according to the recommendations by the 2015-2020 Dietary Guidelines for Americans) (3) and having a healthy diet. The healthy diet component was based on official French nutrition guidelines through an already established score called the PNNS-GS. The PNNS-GS measures adherence to the French recommendations for physical activity and consumption of fruits and vegetables, starchy foods, whole grains, dairy products, meats, seafood, added fat, sweets, water, soda, and salt. It also penalizes for excessive energy consumption, and further details on its construction can be found in previous work (4). The original version of the PNNS-GS contains components on alcohol consumption and physical activity. In order not to take into account these components twice, they were removed from the PNNS-GS score before dichotomizing it in order to create the “healthy diet” component of the HLI. Individuals were attributed the point if they scored above the median value of 6.5 of this modified PNNS-GS ranging from 0 to 12.5.

1. Aleksandrova K, Pischon T, Jenab M, Bueno-de-Mesquita HB, Fedirko V, Norat T, et al. Combined impact of healthy lifestyle factors on colorectal cancer: a large European cohort study. BMC Med. 2014 Oct 10;12:168.
2. WHO | Physical status: the use and interpretation of anthropometry. WHO. Available at: http://www.who.int/childgrowth/publications/physical_status/en/. Accessed May 29, 2017.
3. U.S. Department of Health and Human Services and U.S. Department of Agriculture. 2015 – 2020 Dietary Guidelines for Americans. 8th Edition. December 2015. Available at: <http://health.gov/dietaryguidelines/2015/guidelines/>.
4. Estaquio C, Kesse-Guyot E, Deschamps V, et al. Adherence to the French Programme National Nutrition Santé Guideline Score is associated with better nutrient intake and nutritional status. J Am Diet Assoc. 2009;109(6):1031-1041.

**Supplementary Table 2. Criteria for healthy aging**

| Criteria^a^ | Test or questionnaire | Cut-off | Additional information on the test used |
| --- | --- | --- | --- |
| Good physical functioning | SPPB | ≥11/12 | Physical test battery performed by qualified physicians (such as repeated chair stands, balance testing, and gait speed testing) |
| Good cognitive functioning | MMSE  RI-48  DK-TMT | ≥27/30  ≥19/48  ≥5·5 | Cognitive test battery performed qualified physicians (evaluation of overall cognitive functioning, verbal episodic memory and executive function) |
| No IADL limitations | IADL | <1 limitation | Self-administered questionnaire (questions on the ability to travel, go shopping and do house chores) |
| No depressive symptoms | CES-D | <16/60 | Self-administered questionnaire developed for the evaluation of depressive symptoms in the general population in epidemiological studies |
| No health related limitations in social life | SF-36 item 6 and  SF-36 item 10 | 1-2 and  3–5 | SF-36: self-administered questionnaire used to measure vitality, physical functioning, bodily pain, general health perceptions, physical role functioning, emotional role functioning, social role functioning and mental health |
| Good overall self-perceived health | SF-36 item 1 | 1–3 | SF-36: see above explanations |
| No function limiting pain | SF-36 item 7 and  SF-36 item 8 | 1–3 and  1–2 | SF-36: see above explanations |
| Absence of chronic diseases |  | Absence of cancer (i.e. cancer of any kind, except for basal cell carcinoma), CVD^b^ and diabetes at follow-up | Validation of events by an independent professional committee. No fasting blood glucose value ≥1·26 g/l, use of anti-diabetic medication or presence of self-reported diabetes at the end of follow-up |

Abbreviations: SPPB, Short Physical Performance Battery ; MMSE, Mini Mental State Evaluation ; RI-48, Rappel indicé 48 items; DK-TMT, Delis-Kaplan version of the trail making test; IADL, Instrumental Activities of Daily Living; CES-D, Center for Epidemiologic Studies Depression Scale; SF-36, Medical Outcome Short Study Form-36;

^a^All criteria were evaluated at follow-up (2007-2009), except in the case of major events of chronic diseases which were assessed over the whole duration of the follow-up (1994-2009). Test batteries were distributed in visit centers in hospitals close to participants’ homes and the questionnaires were filled out by the participants at home and certified by technicians. At inclusion, all subjects were free of chronic diseases.

^b^CVD was defined as codes I20–I25, I63, I65, I66, I70, I71 and I74 from the 10th International World Health Organization Classification of Diseases.

|  | HLI=2 | HLI=3 | HLI=4 | HLI=5 | P trend^b^ |
| --- | --- | --- | --- | --- | --- |
|  | **RR 95%CI** | **RR 95%CI** | **RR 95%CI** | **RR 95%CI** |  |
| HLI original |  |  |  |  |  |
| Model 1^c^ | 1.02(0.80, 1.28) | 1.20(0.96, 1.49) | 1.34(1.07, 1.67) | 1.46(1.14, 1.87) | <.001 |
| Model 2^d^ | 1.00(0.79, 1.26) | 1.17(0.94, 1.45) | 1.29(1.03, 1.60) | 1.41(1.10, 1.80) | <.001 |
| Without BMI |  |  |  |  |  |
| Model 1^e^ | 1.13(0.96, 1.33) | 1.22(1.04, 1.44) | 1.38(1.15, 1.66) | _ | <.001 |
| Model 2^f^ | 1.12(0.95, 1.32) | 1.21(1.03, 1.42) | 1.37(1.15, 1.65) | _ | <.001 |
| Model 3^g^ | 1.12(0.95, 1.31) | 1.20(1.02, 1.41) | 1.36(1.14, 1.63) | _ | <.001 |
| Without physical activity |  |  |  |  |  |
| Model 1^e^ | 0.95(0.79, 1.14) | 1.21(1.02, 1.43) | 1.26(1.05, 1.53) | _ | 0.001 |
| Model 2^f^ | 0.93(0.77, 1.11) | 1.15(0.97, 1.37) | 1.19(0.99, 1.44) | _ | 0.01 |
| Model 3^g^ | 0.93(0.77, 1.11) | 1.15(0.97, 1.36) | 1.19(0.99, 1.44) | _ | 0.01 |
| Without smoking status |  |  |  |  |  |
| Model 1^e^ | 1.13(0.98, 1.30) | 1.26(1.10, 1.46) | 1.40(1.17, 1.67) | _ | <.001 |
| Model 2^f^ | 1.11(0.97, 1.28) | 1.23(1.07, 1.42) | 1.37(1.15, 1.63) | _ | <.001 |
| Model 3^g^ | 1.11(0.96, 1.28) | 1.23(1.07, 1.42) | 1.37(1.15, 1.63) | _ | <.001 |
| Without alcohol |  |  |  |  |  |
| Model 1^e^ | 1.27(1.05, 1.54) | 1.42(1.18, 1.71) | 1.66(1.36, 2.02) | _ | <.001 |
| Model 2^f^ | 1.25(1.04, 1.51) | 1.37(1.14, 1.65) | 1.59(1.30, 1.93) | _ | <.001 |
| Model 3^g^ | 1.25(1.04, 1.51) | 1.37(1.14, 1.65) | 1.58(1.30, 1.93) | _ | <.001 |
| Without diet |  |  |  |  |  |
| Model 1^e^ | 1.12(0.94, 1.33) | 1.26(1.06, 1.49) | 1.31(1.08, 1.60) | _ | 0.002 |
| Model 2^f^ | 1.11(0.93, 1.32) | 1.24(1.05, 1.48) | 1.31(1.08, 1.59) | _ | 0.002 |
| Model 3^g^ | 1.11(0.93, 1.32) | 1.25(1.05, 1.48) | 1.30(1.07, 1.58) | _ | 0.003 |

**Supplementary Table 3. Association between the HLI (by scores) and healthy aging (N=2,203)^a^**

Abbreviations: HLI, Healthy Lifestyle Index; BMI, Body Mass Index
^a^Values are RR (95%CI) with HLI= 0 or 1 as the reference
^b^P for linear contrast
^c^Adjusted for age and gender
^d^Adjusted for age, gender, marital status, education, occupational status, supplementation group, number of 24-h dietary records, follow up time and energy intake
^e^Adjusted for all variables in model 1 and removed component
fAdjusted for all variables in model 2 and removed component
^g^Adjusted for all variables in model 2 and more precise removed component

**Supplementary Table 4. HLI in relation to healthy aging for individuals with ≥6 24-h records (N=1,861)^a^**

|  | HLI= 0 or 1 | HLI= 2 | | HLI= 3 | | HLI= 4 | HLI=5 | | P trend^b^ | | Continuous HLI | P^d^ |
| --- | --- | --- | --- | --- | --- | --- | --- | --- | --- | --- | --- | --- |
| HLI original |  |  |  | |  | | |  | |  |  |  |
| Model 1^d^ | 1 _ | 0.99 (0.77, 1.28) | 1.14 (0.90, 1.45) | | 1.32 (1.03, 1.68) | | | 1.40 (1.07, 1.83) | | 0.0008 | 1.11 (1.06, 1.17) | <.0001 |
| Model 2^e^ | 1 _ | 0.98 (0.76, 1.26) | 1.12 (0.88, 1.42) | | 1.27 (1.00, 1.62) | | | 1.37 (1.05, 1.79) | | 0.0016 | 1.11 (1.05, 1.16) | <.0001 |
| Without BMI |  |  |  | |  | | |  | |  |  |  |
| Model 1^f^ | 1 _ | 1.13 (0.94, 1.35) | 1.23 (1.03, 1.46) | | 1.38 (1.14, 1.68) | | | _ | | 0.0005 | 1.11 (1.05, 1.17) | 0.0005 |
| Model 2^g^ | 1 _ | 1.11 (0.93, 1.32) | 1.21 (1.01, 1.44) | | 1.38 (1.14, 1.67) | | | _ | | 0.0006 | 1.10 (1.04, 1.17) | 0.0008 |
| Without physical activity |  |  |  | |  | | |  | |  |  |  |
| Model 1^f^ | 1 _ | 0.91 (0.75, 1.11) | 1.19 (0.99, 1.43) | | 1.20 (0.98, 1.47) | | | _ | | 0.0115 | 1.10 (1.04, 1.17) | 0.001 |
| Model 2^g^ | 1 _ | 0.90 (0.74, 1.09) | 1.13 (0.94, 1.36) | | 1.15 (0.94, 1.41) | | | _ | | 0.0449 | 1.09 (1.02, 1.15) | 0.008 |
| Without smoking status |  |  |  | |  | | |  | |  |  |  |
| Model 1^f^ | 1 _ | 1.12 (0.96, 1.31) | 1.26 (1.08, 1.47) | | 1.38 (1.15, 1.66) | | | _ | | 0.0002 | 1.10 (1.05, 1.16) | 0.0002 |
| Model 2^g^ | 1 _ | 1.11 (0.95, 1.29) | 1.22 (1.05, 1.43) | | 1.36 (1.13, 1.64) | | | _ | | 0.0005 | 1.10 (1.04, 1.16) | 0.0006 |
| Without alcohol |  |  |  | |  | | |  | |  |  |  |
| Model 1^f^ | 1 _ | 1.20 (0.97, 1.47) | 1.35 (1.11, 1.66) | | 1.61 (1.30, 1.99) | | |  | | <.0001 | 1.16 (1.10, 1.23) | <.0001 |
| Model 2^g^ | 1 _ | 1.19 (0.98, 1.46) | 1.33 (1.09, 1.62) | | 1.56 (1.26, 1.92) | | | _ | | <.0001 | 1.15 (1.08, 1.22) | <.0001 |
| Without diet quality |  |  |  | |  | | |  | |  |  |  |
| Model 1^f^ | 1 _ | 1.08 (0.89, 1.30) | 1.23 (1.02, 1.48) | | 1.29 (1.05, 1.59) | | | _ | | 0.0056 | 1.10 (1.03, 1.17) | 0.002 |
| Model 2^g^ | 1 _ | 1.08 (0.89, 1.31) | 1.22 (1.02, 1.47) | | 1.31 (1.06, 1.61) | | | _ | | 0.0041 | 1.10 (1.04, 1.17) | 0.002 |

Abbreviations: HLI, Healthy Lifestyle Index; BMI, Body Mass Index ^a^Values are RR (95%CI)
^b^P for linear contrast
^c^P for the HLI as a continuous variable
^d^Adjusted for age and gender
^e^Adjusted for age, gender, marital status, education, occupational status, supplementation group, number of 24-h dietary records, follow up time and energy intake
^f^Adjusted for all variables in model 1 and removed component
^g^Adjusted for all variables in model 2 and removed component

**Supplementary Figure 1. Flowchart of the selection process**

1,277: Excluded due to one or more missing variables contributing to the definition of “healthy aging”

Participants of the SU.VI.MAX study
N=13,017

6,167: Excluded because not part of the SU.VI.MAX 2 study

6,850: Included in the SU.VI.MAX 2 study observational follow-up study

5,583: Within desired age-range of 45-60 years at baseline

1,267: Excluded because age outside the desired range

5,243: Free of major chronic diseases at baseline

340: Excluded due to diabetes at baseline or event of ischemic disease or cancer before baseline

2,255: Available information for the HLI

1,711: Excluded due to one or more missing variables for the determination of the HLI

2,203: Available information on all covariables

3,966: Available information for the determination of “healthy aging”

52: Excluded due to one or more missing covariables
