# Supplementary material for: How Healthy Lifestyle Factors at Midlife Relate to Healthy Aging
Source: Nutrients. 2018 Jun 30;10(7):854. doi: 10.3390/nu10070854 (PMC6073192; doi:10.3390/nu10070854)
Supplement: Supplementary file 1 [file nutrients-10-00854-s001.zip › Supplementary Table 1.docx]

**Supplementary Table 1. Definition of the HLI**

| Component | Points | Description |
| --- | --- | --- |
| Healthy weight | 0  1 | Overweight or obesity BMI ≥ 25 18.5 ≤ BMI <25 |
| Smoking status | 0  1 | Smoker Former or non-smoker |
| Physical activity | 0  1 | Physically inactive Moderate to high physical activity (>30 min/d brisk walking or equivalent) |
| Alcohol consumption | 0  1 | > 12g/d for women or >24g/j for men ≤ 12g/d for women or ≤ 24g/j for men |
| Diet quality | 0  1 | ≤ 6,5 points on the mPNNS-GS > 6,5 points on the mPNNS-GS |

Abbreviation: mPNNS-GS, Modified Programme National Nutrition Santé-Guideline Score
PNNS-GS measures adherence to the French recommendations for consumption of fruits and vegetables, starchy foods, whole grains, dairy products, meats, seafood, added fat, sweets, water, soda, and salt. It also penalizes for excessive energy consumption. In our study a modified score excluding physical activity and alcohol has been considered (maximum score=12.5).
