# Supplementary material for: How Healthy Lifestyle Factors at Midlife Relate to Healthy Aging
Source: Nutrients. 2018 Jun 30;10(7):854. doi: 10.3390/nu10070854 (PMC6073192; doi:10.3390/nu10070854)
Supplement: Supplementary file 1 [file nutrients-10-00854-s001.zip › Supplementary Table 2.docx]

**Supplementary Table 2. Criteria for healthy aging**

| Criteria^a^ | Test or questionnaire | Cut-off | Additional information on the test used |
| --- | --- | --- | --- |
| Good physical functioning | SPPB | ≥11/12 | Physical test battery performed by qualified physicians (such as repeated chair stands, balance testing, and gait speed testing) |
| Good cognitive functioning | MMSE  RI-48  DK-TMT | ≥27/30  ≥19/48  ≥5·5 | Cognitive test battery performed qualified physicians (evaluation of overall cognitive functioning, verbal episodic memory and executive function) |
| No IADL limitations | IADL | <1 limitation | Self-administered questionnaire (questions on the ability to travel, go shopping and do house chores) |
| No depressive symptoms | CES-D | <16/60 | Self-administered questionnaire developed for the evaluation of depressive symptoms in the general population in epidemiological studies |
| No health related limitations in social life | SF-36 item 6 and  SF-36 item 10 | 1-2 and  3–5 | SF-36: self-administered questionnaire used to measure vitality, physical functioning, bodily pain, general health perceptions, physical role functioning, emotional role functioning, social role functioning and mental health |
| Good overall self-perceived health | SF-36 item 1 | 1–3 | SF-36: see above explanations |
| No function limiting pain | SF-36 item 7 and  SF-36 item 8 | 1–3 and  1–2 | SF-36: see above explanations |
| Absence of chronic diseases |  | Absence of cancer (i.e. cancer of any kind, except for basal cell carcinoma), CVD^b^ and diabetes at follow-up | Validation of events by an independent professional committee. No fasting blood glucose value ≥1·26 g/l, use of anti-diabetic medication or presence of self-reported diabetes at the end of follow-up |

Abbreviations: SPPB, Short Physical Performance Battery ; MMSE, Mini Mental State Evaluation ; RI-48, Rappel indicé 48 items; DK-TMT, Delis-Kaplan version of the trail making test; IADL, Instrumental Activities of Daily Living; CES-D, Center for Epidemiologic Studies Depression Scale; SF-36, Medical Outcome Short Study Form-36;

^a^All criteria were evaluated at follow-up (2007-2009), except in the case of major events of chronic diseases which were assessed over the whole duration of the follow-up (1994-2009). Test batteries were distributed in visit centers in hospitals close to participants’ homes and the questionnaires were filled out by the participants at home and certified by technicians. At inclusion, all subjects were free of chronic diseases.

^b^CVD was defined as codes I20–I25, I63, I65, I66, I70, I71 and I74 from the 10th International World Health Organization Classification of Diseases.
