# Supplementary material for: How Healthy Lifestyle Factors at Midlife Relate to Healthy Aging
Source: Nutrients. 2018 Jun 30;10(7):854. doi: 10.3390/nu10070854 (PMC6073192; doi:10.3390/nu10070854)
Supplement: Supplementary file 1 [file nutrients-10-00854-s001.zip › Supplementary Table 3.docx]

|  | HLI=2 | HLI=3 | HLI=4 | HLI=5 | P trend^b^ |
| --- | --- | --- | --- | --- | --- |
|  | **RR 95%CI** | **RR 95%CI** | **RR 95%CI** | **RR 95%CI** |  |
| HLI original |  |  |  |  |  |
| Model 1^c^ | 1.02(0.80, 1.28) | 1.20(0.96, 1.49) | 1.34(1.07, 1.67) | 1.46(1.14, 1.87) | <.001 |
| Model 2^d^ | 1.00(0.79, 1.26) | 1.17(0.94, 1.45) | 1.29(1.03, 1.60) | 1.41(1.10, 1.80) | <.001 |
| Without BMI |  |  |  |  |  |
| Model 1^e^ | 1.13(0.96, 1.33) | 1.22(1.04, 1.44) | 1.38(1.15, 1.66) | _ | <.001 |
| Model 2^f^ | 1.12(0.95, 1.32) | 1.21(1.03, 1.42) | 1.37(1.15, 1.65) | _ | <.001 |
| Model 3^g^ | 1.12(0.95, 1.31) | 1.20(1.02, 1.41) | 1.36(1.14, 1.63) | _ | <.001 |
| Without physical activity |  |  |  |  |  |
| Model 1^e^ | 0.95(0.79, 1.14) | 1.21(1.02, 1.43) | 1.26(1.05, 1.53) | _ | 0.001 |
| Model 2^f^ | 0.93(0.77, 1.11) | 1.15(0.97, 1.37) | 1.19(0.99, 1.44) | _ | 0.01 |
| Model 3^g^ | 0.93(0.77, 1.11) | 1.15(0.97, 1.36) | 1.19(0.99, 1.44) | _ | 0.01 |
| Without smoking status |  |  |  |  |  |
| Model 1^e^ | 1.13(0.98, 1.30) | 1.26(1.10, 1.46) | 1.40(1.17, 1.67) | _ | <.001 |
| Model 2^f^ | 1.11(0.97, 1.28) | 1.23(1.07, 1.42) | 1.37(1.15, 1.63) | _ | <.001 |
| Model 3^g^ | 1.11(0.96, 1.28) | 1.23(1.07, 1.42) | 1.37(1.15, 1.63) | _ | <.001 |
| Without alcohol |  |  |  |  |  |
| Model 1^e^ | 1.27(1.05, 1.54) | 1.42(1.18, 1.71) | 1.66(1.36, 2.02) | _ | <.001 |
| Model 2^f^ | 1.25(1.04, 1.51) | 1.37(1.14, 1.65) | 1.59(1.30, 1.93) | _ | <.001 |
| Model 3^g^ | 1.25(1.04, 1.51) | 1.37(1.14, 1.65) | 1.58(1.30, 1.93) | _ | <.001 |
| Without diet |  |  |  |  |  |
| Model 1^e^ | 1.12(0.94, 1.33) | 1.26(1.06, 1.49) | 1.31(1.08, 1.60) | _ | 0.002 |
| Model 2^f^ | 1.11(0.93, 1.32) | 1.24(1.05, 1.48) | 1.31(1.08, 1.59) | _ | 0.002 |
| Model 3^g^ | 1.11(0.93, 1.32) | 1.25(1.05, 1.48) | 1.30(1.07, 1.58) | _ | 0.003 |

**Supplementary Table 3. Association between the HLI (by scores) and healthy aging (N=2,203)^a^**

Abbreviations: HLI, Healthy Lifestyle Index; BMI, Body Mass Index
^a^Values are RR (95%CI) with HLI= 0 or 1 as the reference
^b^P for linear contrast
^c^Adjusted for age and gender
^d^Adjusted for age, gender, marital status, education, occupational status, supplementation group, number of 24-h dietary records, follow up time and energy intake
^e^Adjusted for all variables in model 1 and removed component
fAdjusted for all variables in model 2 and removed component
^g^Adjusted for all variables in model 2 and more precise removed componen
