# Supplementary material for: How Healthy Lifestyle Factors at Midlife Relate to Healthy Aging
Source: Nutrients. 2018 Jun 30;10(7):854. doi: 10.3390/nu10070854 (PMC6073192; doi:10.3390/nu10070854)
Supplement: Supplementary file 1 [file nutrients-10-00854-s001.zip › Supplementary Table 4.docx]

**Supplementary Table 4. HLI in relation to healthy aging for individuals with ≥6 24-h records (N=1,861)^a^**

|  | HLI= 0 or 1 | HLI= 2 | | HLI= 3 | | HLI= 4 | HLI=5 | | P trend^b^ | | Continuous HLI | P^d^ |
| --- | --- | --- | --- | --- | --- | --- | --- | --- | --- | --- | --- | --- |
| HLI original |  |  |  | |  | | |  | |  |  |  |
| Model 1^d^ | 1 _ | 0.99 (0.77, 1.28) | 1.14 (0.90, 1.45) | | 1.32 (1.03, 1.68) | | | 1.40 (1.07, 1.83) | | 0.0008 | 1.11 (1.06, 1.17) | <.0001 |
| Model 2^e^ | 1 _ | 0.98 (0.76, 1.26) | 1.12 (0.88, 1.42) | | 1.27 (1.00, 1.62) | | | 1.37 (1.05, 1.79) | | 0.0016 | 1.11 (1.05, 1.16) | <.0001 |
| Without BMI |  |  |  | |  | | |  | |  |  |  |
| Model 1^f^ | 1 _ | 1.13 (0.94, 1.35) | 1.23 (1.03, 1.46) | | 1.38 (1.14, 1.68) | | | _ | | 0.0005 | 1.11 (1.05, 1.17) | 0.0005 |
| Model 2^g^ | 1 _ | 1.11 (0.93, 1.32) | 1.21 (1.01, 1.44) | | 1.38 (1.14, 1.67) | | | _ | | 0.0006 | 1.10 (1.04, 1.17) | 0.0008 |
| Without physical activity |  |  |  | |  | | |  | |  |  |  |
| Model 1^f^ | 1 _ | 0.91 (0.75, 1.11) | 1.19 (0.99, 1.43) | | 1.20 (0.98, 1.47) | | | _ | | 0.0115 | 1.10 (1.04, 1.17) | 0.001 |
| Model 2^g^ | 1 _ | 0.90 (0.74, 1.09) | 1.13 (0.94, 1.36) | | 1.15 (0.94, 1.41) | | | _ | | 0.0449 | 1.09 (1.02, 1.15) | 0.008 |
| Without smoking status |  |  |  | |  | | |  | |  |  |  |
| Model 1^f^ | 1 _ | 1.12 (0.96, 1.31) | 1.26 (1.08, 1.47) | | 1.38 (1.15, 1.66) | | | _ | | 0.0002 | 1.10 (1.05, 1.16) | 0.0002 |
| Model 2^g^ | 1 _ | 1.11 (0.95, 1.29) | 1.22 (1.05, 1.43) | | 1.36 (1.13, 1.64) | | | _ | | 0.0005 | 1.10 (1.04, 1.16) | 0.0006 |
| Without alcohol |  |  |  | |  | | |  | |  |  |  |
| Model 1^f^ | 1 _ | 1.20 (0.97, 1.47) | 1.35 (1.11, 1.66) | | 1.61 (1.30, 1.99) | | |  | | <.0001 | 1.16 (1.10, 1.23) | <.0001 |
| Model 2^g^ | 1 _ | 1.19 (0.98, 1.46) | 1.33 (1.09, 1.62) | | 1.56 (1.26, 1.92) | | | _ | | <.0001 | 1.15 (1.08, 1.22) | <.0001 |
| Without diet quality |  |  |  | |  | | |  | |  |  |  |
| Model 1^f^ | 1 _ | 1.08 (0.89, 1.30) | 1.23 (1.02, 1.48) | | 1.29 (1.05, 1.59) | | | _ | | 0.0056 | 1.10 (1.03, 1.17) | 0.002 |
| Model 2^g^ | 1 _ | 1.08 (0.89, 1.31) | 1.22 (1.02, 1.47) | | 1.31 (1.06, 1.61) | | | _ | | 0.0041 | 1.10 (1.04, 1.17) | 0.002 |

Abbreviations: HLI, Healthy Lifestyle Index; BMI, Body Mass Index ^a^Values are RR (95%CI)
^b^P for linear contrast
^c^P for the HLI as a continuous variable
^d^Adjusted for age and gender
^e^Adjusted for age, gender, marital status, education, occupational status, supplementation group, number of 24-h dietary records, follow up time and energy intake
^f^Adjusted for all variables in model 1 and removed component
^g^Adjusted for all variables in model 2 and removed component
