# Supplementary material for: How Healthy Lifestyle Factors at Midlife Relate to Healthy Aging
Source: Nutrients. 2018 Jun 30;10(7):854. doi: 10.3390/nu10070854 (PMC6073192; doi:10.3390/nu10070854)
Supplement: Supplementary file 1 [file nutrients-10-00854-s001.zip › Supplementary Text 1.docx]

**Supplementary Text 1. Computation of the Healthy Lifestyle Index**

The Healthy Lifestyle Index (HLI) was based on a previous study (1) and included risk factors often associated with age-related chronic diseases and functioning disorders. The index was constituted of five components, each allocated a binary score. A score of 1 was attributed to favorable behaviors, while a score of 0 was allocated when conditions were not met. The final score was obtained by summing the scores of each individual component. Thus, the HLI ranged from 0 (for least healthy) to 5 (for most healthy). The index took into account having a healthy weight (18.5<BMI<25 according to the WHO cutoff points)(2), being a non-smoker (former or non-smoker), being physically active (an activity equivalent to at least 30 min of brisk walking per day), being a moderate drinker (up to 2 standard drinks/d for men and up to 1 standard drink/d for women according to the recommendations by the 2015-2020 Dietary Guidelines for Americans) (3) and having a healthy diet. The healthy diet component was based on official French nutrition guidelines through an already established score called the PNNS-GS. The PNNS-GS measures adherence to the French recommendations for physical activity and consumption of fruits and vegetables, starchy foods, whole grains, dairy products, meats, seafood, added fat, sweets, water, soda, and salt. It also penalizes for excessive energy consumption, and further details on its construction can be found in previous work (4). The original version of the PNNS-GS contains components on alcohol consumption and physical activity. In order not to take into account these components twice, they were removed from the PNNS-GS score before dichotomizing it in order to create the “healthy diet” component of the HLI. Individuals were attributed the point if they scored above the median value of 6.5 of this modified PNNS-GS ranging from 0 to 12.5.

1. Aleksandrova K, Pischon T, Jenab M, Bueno-de-Mesquita HB, Fedirko V, Norat T, et al. Combined impact of healthy lifestyle factors on colorectal cancer: a large European cohort study. BMC Med. 2014 Oct 10;12:168.
2. WHO | Physical status: the use and interpretation of anthropometry. WHO. Available at: http://www.who.int/childgrowth/publications/physical_status/en/. Accessed May 29, 2017.
3. U.S. Department of Health and Human Services and U.S. Department of Agriculture. 2015 – 2020 Dietary Guidelines for Americans. 8th Edition. December 2015. Available at: <http://health.gov/dietaryguidelines/2015/guidelines/>.
4. Estaquio C, Kesse-Guyot E, Deschamps V, et al. Adherence to the French Programme National Nutrition Santé Guideline Score is associated with better nutrient intake and nutritional status. J Am Diet Assoc. 2009;109(6):1031-1041.
